# Prevalence of Depressive Symptoms and Its Associated Factors among People Living with HIV Attending Public Hospitals of Nekemte Town, Western Ethiopia, 2021

**DOI:** 10.1155/2021/8854791

**Published:** 2021-07-01

**Authors:** Eba Abdisa, Tizita Tolesa, Muktar Abadiga

**Affiliations:** ^1^School of Nursing and Midwifery, Institute of Health Sciences, Wollega University, Nekemte, Ethiopia; ^2^Ambo University Referral Hospital, Ambo University, Ambo, Ethiopia

## Abstract

**Background:**

Depressive symptoms are the most common mental illness among people living with HIV/AIDS. Depressive symptoms impact negatively on the course of HIV infection and can lead to suicide and increased risk of mortality when it is a severe form. Although depressive symptoms are common among HIV/AIDS patients, only a few studies have been conducted in Ethiopia and no study, particularly at Nekemte town public hospitals. Therefore, this study was aimed at assessing the prevalence and risk factors for depressive symptoms among people living with HIV/AIDS attending Nekemte town public hospitals, Western Ethiopia*. Methods*. An institution-based cross-sectional study design was conducted on 425 HIV/AIDS patients at Nekemte town public hospitals, from March 30 to May 30, 2019. Data were collected through interviews and patient document reviews. The nine-item Patient Health Questionnaire (PHQ-9) was used to collect information concerning depressive symptoms and was defined by a PHQ-9 score ≥ 5. HIV stigma and discrimination scales were used to measure stigma. Social support was described by a sum score of the Oslo3 social support scale (OSS-3). The collected data was entered into EpiData Windows version 4.1 and then exported to Statistical Package for the Social Sciences (SPSS) Windows version 24.0 for analysis. All variables found to be significant at the bivariable level (*p* value < 0.25) were entered into a multivariable logistic regression model. *p* values of <0.05 and 95% confidence level were used to determine statistical significance.

**Results:**

Out of the total of 384 study participants who participated in the study, 165 (42.96%) had depressive symptoms. Self-reported sleeping problems (AOR = 7.04, 95% CI: 3.23, 15.33), CD4 level of <200 (AOR = 5.45, 95% CI: 2.06, 14.42), poor social support (AOR = 2.79, 95% CI: 1.17, 6.67), and perceived stigma (AOR = 9.11, 95% CI: 1.17, 17.33) were significantly associated with depressive symptoms among HIV/AIDS patients at Nekemte town public hospitals.

**Conclusion:**

The level of depressive symptoms among HIV/AIDS patients in this study was high. Self-reported sleeping problems, CD4 level, social support, and perceived stigma were found to be significantly associated with depressive symptoms among HIV patients. Health care professionals should have to strengthen the linkage of mental health with antiretroviral therapy (ART) clinic to early detect and treat depressive symptoms.

## 1. Introduction

Depressive symptoms are one of the most common mental illnesses worldwide, affecting approximately 121 million people and accounting for 5% of disability-adjusted life years [1]. It is characterized by sadness, loss of interest or pleasure, feelings of guilt or low self-worth, disturbed sleep or appetite, feelings of tiredness, and poor concentration [2]. Depressive symptoms are the most common mental illness among people living with HIV/AIDS. However, half of all PLHIV with depressive symptoms go underdiagnosed and untreated [3].

The proportion of PLHIV with depressive symptoms is particularly high in developing countries [4]. Studies conducted in different countries on prevalence of depressive symptoms among HIV patients showed 40.9% in China [5], 58.75% in India [6], 29.4% in Brazil [7], 56.7% in Nigeria [8], 18.9% in Malawi [9], 26.7% in Cameroon [10], and 47% in Uganda [11]. For depressive symptoms in Ethiopia, studies have emerged from different regions of the country. The prevalence of depressive symptoms among PLHIVs was 43.9% in Tigray region [12], 41.7% in Gimbi hospital [13], 45.8% in Harar [14], 14.6% in Axum [15], 32% in Hawassa [16], and 38.94% in Debre Birhan [17].

Depressive symptoms impact negatively on the course of HIV infection and can lead to suicide and increased risk of mortality when it is a severe form [18, 19]. Depressive symptoms often run a chronic course and impair an individual's occupational potential and quality of life [20, 21]. Depressive symptom occurrence in PLHIV leads to alteration of economic productivity, a decrease in working ability, social isolation, physical decline and difficulties in solving problems, repetitive negative thinking, and increased forgetting or deficits in concentration which are again more severe in PLHIV [22]. Depressive symptoms may hurt the quality of life of PLHIV, reduce medication adherence, and increase AIDS disease progression [23]. The outcome of HIV among mentally ill subjects has been observed to be poorer which might be attributed to several factors like poor adherence to highly active antiretroviral therapy (HAART), lower accessibility to HAART, and immunological changes associated with the mental illness itself [24].

The studies showed that depressive symptoms are associated with different sociodemographic and health-related factors such as age, sex, marital status, monthly income, occupational status, opportunistic infection, adverse drug reaction, and presence of other chronic diseases [12–14]. In addition, other important factors such as social stigma, occupational disability, isolation from social support, long-term physical discomfort, and illness have been attributed to the occurrence of depressive symptoms on PLHIV [25].

Although the prevalence of depressive symptoms is high among HIV/AIDS patients, there was no published study, particularly at Nekemte town public hospitals. The prevalence and risk factors for depressive symptoms may vary from place to place based on variation in culture, level of income, and geographical distribution. This study was done to fill this research gap which might provide evidence for the future's effective prevention and treatment of depressive symptoms in people living with HIV/AIDS. Therefore, this study was aimed at assessing the prevalence and risk factors for depressive symptoms among people living with HIV/AIDS attending Nekemte town public hospitals, Western Ethiopia. The findings of this study will improve awareness of health care providers related to depressive symptoms among PLHIV and provide evidence for providing patient-centered nursing care.

## 2. Materials and Methods

### 2.1. Study Setting and Population

An institution-based cross-sectional study design was conducted in Nekemte town public hospitals, Western Ethiopia, from March 30 to May 30, 2019. Nekemte town is the capital of East Wollega Zone located 321 km from Addis Ababa to the west. Administratively, the town is divided into six subcity administrations or 12 kebeles. The total population of the town is projected to be 97,289 of which 38,385 (51%) were females [26]. Nekemte town has two governmental public hospitals, namely, Nekemte Specialized Hospital and Wollega University specialized hospital. There were 2124 PLHIV attending ART clinics in both hospitals during the time of the study. All HIV-infected patients attending ART at Nekemte town public hospitals were the source population, and sampled HIV-infected patients attending ART at Nekemte town public hospitals were the study population. All HIV-positive patients attending ART with the age of 18 and above and HIV-positive patients who offered consent and signed the agreement form were included in the study. Severely ill patients who cannot give a response and are unable to communicate were excluded from the study.

### 2.2. Sample Size Determination and Sampling Techniques

The sample size for this study was calculated using a formula for estimation of single population proportion with the assumption of 95% confidence level and 4.5% margin of error and by taking 45.8% prevalence of depressive symptoms from a previous study conducted in Harar town [14]. After adding a 10% nonresponse rate, 425 HIV/AIDS patients were enrolled in the study. The sample size was proportionally allocated to both hospitals based on the number of clients who received service in the previous month. At each site, participants fulfilling inclusion criteria were selected using systematic random sampling methods. Data were collected in every 5^th^ interval until the desired sample became achieved in each hospital identifying initial starting participants by using a lottery method.

### 2.3. Data Collection Tool and Procedure

Data were collected through interview and document review (for CD4 level and opportunistic infection (OI), stages of HIV, and the side effect of the drug-related data) using a checklist and structured questionnaire. The checklist was prepared for CD4 level and opportunistic infection (OI), stages of HIV, and side effects of the drug. The nine-item Patient Health Questionnaire (PHQ-9) was used to collect information concerning depressive symptoms and was defined by a PHQ-9 score ≥ 5 [27]. The level of depressive symptoms among HIV/AIDS clients was measured using a nine-item Patient Health Questionnaire (PHQ-9) having a score range from 0 to 27. HIV/AIDS clients who scored PHQ-9 score ≥ 5 were considered depressed, and clients who scored <5 were considered not depressed. HIV stigma and discrimination scales were used to measure stigma [28]. Self-reported sleeping problems were measured by asking the patients whether they have any sleeping difficulties recently. Social support was described by a sum score of the Oslo3 social support scale (OSS-3) as strong social support (12–14), intermediate social support (9–11), and poor social support (3–8) [29]. The patients were asked whether they have any other chronic diseases apart from depression, and the patients were considered having the chronic disease if at least one chronic disease is present. In addition, the patients were asked whether they use alcohol and considered using alcohol if currently using it. Data collections included three BSc nurses and two supervisors. The data was collected from March 30 to May 30, 2019, for about a 2-month period.

### 2.4. Data Processing and Analysis

The collected data were coded on a prearranged coding sheet and entered into EpiData Windows version 4.1 statistical programs. The data was cleaned accordingly and then exported to Statistical Package for the Social Sciences (SPSS) Windows version 24.0 for analysis. Descriptive analyses such as percentages, frequency distribution, and measures of central tendencies were used. Bivariable analysis was performed between a dependent variable and each of the independent variables. Then, all variables found to be significant at the bivariable level (*p* value < 0.25) were entered into a multivariable logistic regression model. *p* values of <0.05 and 95% confidence level were used to determine statistical significance.

### 2.5. Data Control and Management

The questionnaire prepared in English was translated to the local language Afaan Oromoo and retranslated back to English by people who are proficient in both languages to maintain the consistency of the questionnaires. A pretest was carried out with 5% of the sample size in Shambu Hospital which was not included in the study. Two days of intensive training was given for data collectors and supervisors on data collection tools, techniques of interview, and document review. The filled questionnaire was checked for completeness, accuracy, and consistency. Data were cleaned and edited after it is entered into the software.

### 2.6. Ethical Consideration

Ethical clearance and permission were obtained from the Wollega University Institutional Review Board (IRB), and a permission letter was secured from Nekemte Specialized Hospital and Wollega University specialized hospital. Study participants were provided with information about the objectives of the study and the right not to respond fully or partially to the questionnaire, and written informed consent was obtained from each respondent before the interview. Confidentiality of individual client information was ensured by using unique identifiers and limiting access to the third party by storing the completed questionnaires with participant information in a lockable cabinet.

## 3. Results

### 3.1. Sociodemographic Characteristics of the Study Participants

Out of the total study participants (425), 384 respondents were included in the analysis, with a 90.14% response rate. The mean age of the study participants was 36.83 (±SD 10.68), and about 152 (60.4%) of the study participants were females. The majority 324 (84.4%) were Oromo by ethnicity, and half of the participants 203 (52.3%) were protestant religion followers. About one-fourth (26%) of the participants were merchants, and 148 (38.5%) of participants had an income level of less than 500 ETB. Out of the total respondents, 177 (41.6%) participants had other comorbid chronic diseases (Table 1).

### 3.2. HIV Status and Related Clinical Risk Factors

The majority 367 (95.6%) of respondents were in HIV stage I. About two-fifths 151 (39.3%) of the respondents had a CD4 count greater than 500 cells/mm^3^. Opportunistic infections were prevalent in 69 (18%) of the respondents. About 294 (76.6%) of the respondents had started ART before 60 months, and the remaining 23.4% were on ART for a ≤23-month to 59-month duration. Among those on ART, about 143 (37.2%) had experienced drug side effects (Table 2).

### 3.3. Level of Depressive Symptoms among HIV/AIDS Patients on ART Follow-Up

Out of the total of 384 study participants involved in the study, 165 (42.96%) had depressive symptoms and 219 (57.03%) had no depressive symptoms.

### 3.4. Factors Associated with Depressive Symptoms

Binary logistic regression was performed to assess the association of each independent variable with depressive symptoms. The result of bivariable logistic regression analysis showed that sex, educational status, occupational status, income level, presence of chronic disease, alcohol use, duration of HIV/AIDS, family history of mental illness, CD4 level, side effect of the drug, self-reported sleeping problems, change on the feeling of sex, perceived stigma, and social support were associated with depressive symptoms at a *p* value less than 0.25. Variables that showed a *p* value of less than 0.25 in the bivariable analysis were considered the candidate for the multivariable regression model. In the multivariable logistic regression, self-reported sleeping problems, CD4 level, social support, and perceived stigma were significantly associated with depressive symptoms at a *p* value of <0.05. The study participants who had self-reported sleeping problems were about seven times more likely to have depressive symptoms than their counterparts (AOR = 7.04, 95% CI: 3.23, 15.33). Respondents whose CD4 level is <200 were about five times more likely to be depressed than those with a CD4 level of >500 (AOR = 5.45, 95% CI: 2.06, 14.42). Study participants who have poor social support were about 3 times more likely to have depressive symptoms than those who had strong social support (AOR = 2.79, 95% CI: 1.17, 6.67). The study participants who had perceived stigma related to their HIV status were about nine times more likely to develop depressive symptoms than their counterparts (AOR = 9.11, 95% CI: 1.17, 17.33) (Table 3).

## 4. Discussion

In this study, we assessed the prevalence and risk factors for depressive symptoms among HIV/AIDS patients in public hospitals of Nekemte town. The overall prevalence of depressive symptoms among the study participants in this study was 42.9%. This finding is similar to the finding from a study conducted in China [5], Tigray [12], Gimbi [13], and Harar [14]. However, this study finding is higher compared to the study conducted in Kenya [30], Malawi [9], Axum [15], and Hawassa [16]. On the other hand, this finding was lower compared to the study conducted in Uganda [11], Nigeria [8], China [31], and Fiche [32]. This variation could be due to variation in the geographical region, the difference in country income, the usage of different types of tools, sample size, and study population.

In this study, factors such as self-reported sleeping problems, CD4 level, social support, and perceived stigma were found to be significantly associated with depressive symptoms. The finding of this study revealed that participants who have poor social support were about three times more likely to have depressive symptoms than those participants who have strong social support. This might be because poor social support may lead to social isolation which might lead to depressive symptoms. This finding is supported by a study carried out in Hawassa [16], China [31], and Gimbi [13]. However, this finding is not supported by the study conducted in Malawi [9], Axum [15], and Harar [14]. Although social support provides warmth and care and decreases the stress faced by patients, about 53% of patients considered them to be indifferent, hostile, or unsupportive [11].

Self-reported sleeping problems are another important factor for depressive symptoms. The result of this study revealed that study participants with self-reported sleeping problems are about two times more likely to have depressive symptoms than those with no self-reported sleeping problems. This finding is consistent with a study conducted in the USA [33]. On the other hand, a study conducted in China [31], Kenya [30], Malawi [9], Nigeria [8], Hawassa [16], and Gimbi [13] does not support this study finding. These result findings might alert clinicians to assess and provide specific treatment for self-reported sleeping problems to patients with HIV/AIDS as this might lead to depressive symptoms.

The level of CD4 of the participants was another important factor that affects depressive symptoms. In this study, participants with a CD4 level of less than 200 were about four times more likely to be depressed compared to those participants with CD4 of greater than or equal to 500. This finding is in line with a study conducted in Uganda which showed that patients with low CD4 counts were more likely to be depressed [11]. This might be attributed to the likelihood of opportunistic infection that follows a low CD4 level. But the finding is inconsistent with a study from Brazil which showed no statistical difference between the mean of CD4 and depressive symptoms [34].

In this study, perceived stigma due to HIV/AIDS is found to be significantly associated with depressive symptoms. The finding of this study indicated that those participants who perceived social stigma were nine times more likely to have depressive symptoms than those who did not perceive social stigma. This finding is in line with the study conducted in China [5], Gimbi [13], Hawassa [16], Addis Ababa [35], and Gondar [36]. This might be because perceived HIV-related stigma in the community may cause people living with HIV to internalize the stigma and anticipate stigmatizing experiences, resulting in adverse health and psychosocial outcomes like depressive symptoms [37].

### 4.1. Limitation of the Study

The study was cross-sectional and could only determine whether there is an association between depressive symptoms and associated factors and not whether this association is a cause-effect relationship. The study was hospital-based, and as such, some patients with depressive symptom disorders especially patients with severe depressive symptoms were unlikely to present to the hospital.

## 5. Conclusion

This study showed that the level of depressive symptoms among HIV/AIDS patients was high. Factors such as self-reported sleeping problems, CD4 level, social support, and perceived stigma were found to be significantly associated with depressive symptoms among HIV patients. Health care professionals should have to strengthen the linkage of mental health with ART clinics to early detect and treat depressive symptoms.

## Figures and Tables

**Table 1:** Sociodemographic characteristics of PLHIV who were attending ART clinic in Nekemte town public hospitals, Western Ethiopia, 2019.

Variables	Category	Frequency (*n*)	Percent (%)
Age	18-25	58	15.1
	26-35	127	33.1
	36-45	122	31.8
	46-55	60	15.6
	≥55	17	4.4
Sex	Male	152	39.6
	Female	232	60.4
Residence	Urban	331	86.2
	Rural	53	13.8
Marital status	Married	190	49.5
	Single	65	16.9
	Widowed	86	22.4
	Divorced	43	11.2
Education	Unable to read and write	72	18.8
	Primary school	153	39.8
	Secondary school	106	27.6
	Diploma and above	53	13.8
Occupation	Student	36	9.4
	Merchant	100	26.0
	Farmer	23	6.0
	Employer	55	14.3
	Daily laborer	80	20.8
	Housewife	55	14.3
	Unemployed	35	9.1
Religion	Protestant	201	52.3
	Orthodox	141	36.7
	Catholic	32	8.3
	Others	8	2.1
Ethnicity	Oromo	324	84.4
	Amhara	51	13.3
	Others	4	1.0
Income	<500	148	38.5
	500-1000	69	18.0
	1001-2000	85	22.1
	>2000	82	21.4

**Table 2:** HIV/AIDS status-related characteristics among PLHIV on attending ART clinic in Nekemte town public hospitals, Western Ethiopia, 2019.

Variables	Category	Frequency (*n*)	Percent (%)
CD4 count	<200	47	12.2
	200-350	88	22.9
	>350-500	98	25.5
	>500	151	39.3
Opportunistic infections	Yes	69	18.0
	No	315	82.0
Stages of HIV	Stage 1	367	95.6
	Stage 2 and above	8	2.1
Side effect	Yes	143	37.2
	No	241	62.8
ART initiation	≤23 months	30	7.8
	24-59 months	60	15.6
	≥60 months	294	76.6
Family history of mental illness	Yes	66	17.2
	No	318	82.8
History of chronic disease	Yes	177	46.1
	No	207	53.9

**Table 3:** Multivariable analysis of factors associated with depressive symptoms among PLHIV attending ART clinic in Nekemte town public hospitals, Oromia, West Ethiopia, 2019.

Variables	Category	COR with 95% CI	AOR with 95% CI	*p* value
Sex	Female	1.59 (1.05, 2.42)	1.08 (0.54, 2.17)	0.83
	Male	ref	ref	
Education	Cannot read and write	2.173 (1.04, 4.52)	1.55 (0.38, 6.35)	0.55
	Primary	1.44 (0.75, 2.76)	2 (0.56, 7.17)	0.29
	Secondary	1.38 (0.69, 2.74)	1.87 (0.56, 6.2)	0.31
	Diploma and above	ref	ref	
Income	<500	2.21 (1.26, 3.88)	0.48 (0.14, 1.65)	0.24
	500-1000	1.16 (0.59, 2.27)	0.46 (0.1, 1.47)	0.19
	1001-2000	1.50 (0.80, 2.81)	0.49 (0.17, 1.38)	0.18
	>2000	ref	ref	
Occupation	Student	0.67 (0.26, 1.71)	1.20 (0.29, 4.93)	0.8
	Merchant	0.43 (0.19, 0.94)	0.38 (0.1, 1.43)	0.15
	Farmer	0.51 (0.18, 1.49)	0.59 (0.11, 3.14)	0.54
	Employer	0.32 (0.14, 0.78)	0.19 (0.04, 0.91)	0.40
	Daily laborer	0.38 (0.17, 0.86)	0.33 (0.1, 1.12)	0.08
	Housewife	0.8 (0.34, 1.89)	0.48 (0.13, 1.76)	0.27
	Unemployed	ref	ref	
Presence of chronic disease	Yes	2.08 (1.38, 3.13)	1.53 (0.84, 2.78)	0.16
	No	ref	ref	
Duration of HIV	≤23	0.26 (0.1, 0.64)	0.11 (0.01, 0.65)	0.150
	24-59 months	0.55 (0.31, 1.01)	0.11 (0.021, 0.59)	0.10
	≥60 months	ref	ref	
Duration since ART initiation	≤23	ref	ref	
	24-59 months	1.5 (0.617, 3.65)	2.96 (0.31, 27.99)	0.35
	≥60 months	1.08 (0.01, 2.322)	0.34 (0.06, 2.12)	0.25
Alcohol usage	Yes	1.9 (0.99, 3.63)	1.51 (0.56, 4.05)	0.42
	No	ref	ref	
Family history of mental illness	Yes	3.004 (1.72, 5.22)	1.56 (0.67, 3.63)	0.3
	No	ref	ref	
Side effect of medication	Yes	1.85 (1.21, 2.81)	1.56 (0.85, 2.86)	0.15
	No	ref	ref	
Self-reported sleeping problems	Yes	5.263 (3.085, 8.88)	7.04 (3.23, 15.33)	<0.001^∗^
	No	ref	ref	
CD4 level	<200	4.309 (2.12, 8.68)	5.45 (2.06, 14.42)	0.001^∗^
	200-350	1.93 (1.127, 3.306)	1.85 (0.85, 4.03)	0.122
	>350-500	1.39 (0.823, 2.4)	1.70 (0.79, 3.67)	0.173
	>500	ref	ref	
Change on feeling of sex	Yes	3.97 (2.48, 6.34)	4.03 (2.01, 8.05)	0.075
	No	ref	ref	
Social support	Poor	6.64 (3.55, 12.51)	2.79 (1.17, 6.67)	0.021^∗^
	Intermediate	3.95 (2.30, 6.78)	2.09 (1.17, 4.3)	0.450
	Strong	ref	ref	
Perceived stigma	Yes	7.35 (4.66, 11.6)	9.11 (1.17, 17.33)	<0.001^∗^
	No	ref	ref	

∗ indicates significance at *p* value < 0.05. COR: crude odds ratio; AOR: adjusted odds ratio; CI: confidence interval.

## Data Availability

All data are fully available from the corresponding author with reasonable request.

## Abbreviations

AIDS: Acquired immunodeficiency virus
AOR: Adjusted odds ratio
ART: Antiretroviral therapy
CI: Confidence interval
COR: Crude odds ratio
DALYs: Disability-adjusted life years
ETB: Ethiopian birr
HAART: Highly active antiretroviral therapy
HIV: Human immunodeficiency virus
OIs: Opportunistic infections
OSS: Oslo social support scale
PHQ: Patient Health Questionnaire
PLHIV: People living with human immunodeficiency virus
SD: Standard deviation
SPSS: Statistical Package for the Social Sciences
WHO: World Health Organization.
